# Prognostic prediction by a novel integrative inflammatory and nutritional score based on least absolute shrinkage and selection operator in esophageal squamous cell carcinoma

**DOI:** 10.3389/fnut.2022.966518

**Published:** 2022-11-10

**Authors:** Jifeng Feng, Liang Wang, Xun Yang, Qixun Chen, Xiangdong Cheng

**Affiliations:** ^1^The Second Clinical Medical College, Zhejiang Chinese Medical University, Hangzhou, China; ^2^Department of Thoracic Oncological Surgery, Chinese Academy of Science, The Cancer Hospital of the University of Chinese Academy of Sciences (Zhejiang Cancer Hospital), Institute of Basic Medicine and Cancer (IBMC), Hangzhou, China; ^3^Chinese Academy of Science, Zhejiang Provincial Research Center for Upper Gastrointestinal Tract Cancer, Key Laboratory of Prevention, Diagnosis and Therapy of Upper Gastrointestinal Cancer of Zhejiang Province, The Cancer Hospital of the University of Chinese Academy of Sciences (Zhejiang Cancer Hospital), Institute of Basic Medicine and Cancer (IBMC), Hangzhou, China

**Keywords:** least absolute shrinkage and selection operator (LASSO), cancer-specific survival (CSS), esophageal squamous cell carcinoma (ESCC), prognosis, integrative inflammatory and nutritional score

## Abstract

**Background:**

This study aimed to establish and validate a novel predictive model named integrative inflammatory and nutritional score (IINS) for prognostic prediction in esophageal squamous cell carcinoma (ESCC).

**Materials and methods:**

We retrospectively recruited 494 pathologically confirmed ESCC patients with surgery and randomized them into training (*n* = 346) or validation group (*n* = 148). The least absolute shrinkage and selection operator (LASSO) Cox proportional hazards (PH) regression analysis was initially used to construct a novel predictive model of IINS. The clinical features and prognostic factors with hazard ratio (HRs) and 95% confidence intervals (CIs) grouped by IINS were analyzed. Nomogram was also established to verify the prognostic value of IINS.

**Results:**

According to the LASSO Cox PH regression analysis, a novel score of IINS was initially constructed based on 10 inflammatory and nutritional indicators with the optimal cut-off level of 2.35. The areas under the curve (AUCs) of IINS regarding prognostic ability in 1-year, 3-years, and 5-years prediction were 0.814 (95% CI: 0.769–0.854), 0.748 (95% CI: 0.698–0.793), and 0.792 (95% CI: 0.745–0.833) in the training cohort and 0.802 (95% CI: 0.733–0.866), 0.702 (95% CI: 0.621–0.774), and 0.748 (95% CI: 0.670–0.816) in the validation cohort, respectively. IINS had the largest AUCs in the two cohorts compared with other prognostic indicators, indicating a higher predictive ability. A better 5-years cancer-specific survival (CSS) was found in patients with IINS ≤ 2.35 compared with those with IINS > 2.35 in both training cohort (54.3% vs. 11.1%, *P* < 0.001) and validation cohort (53.7% vs. 18.2%, *P* < 0.001). The IINS was then confirmed as a useful independent factor (training cohort: HR: 3.000, 95% CI: 2.254–3.992, *P* < 0.001; validation cohort: HR: 2.609, 95% CI: 1.693–4.020, *P* < 0.001). Finally, an IINS-based predictive nomogram model was established and validated the CSS prediction (training set: C-index = 0.71 and validation set: C-index = 0.69, respectively).

**Conclusion:**

Preoperative IINS is an independent predictor of CSS in ESCC. The nomogram based on IINS may be used as a potential risk stratification to predict individual CSS and guide treatment in ESCC with radical resection.

## Introduction

As one of the most frequent cancers, esophageal cancer (EC) ranks 10th in cancer incidence (0.60 million new diagnosed cases) and 6th in cancer death (0.54 million died cases) in 2020 based on the global cancer statistics (1). EC [mostly esophageal squamous cell carcinoma, (ESCC)] is one of the most common and aggressive cancers in China, with approximately 0.32 million new cases (6th in incidence) and 0.30 million deaths (4th in mortality) in 2020 (2). Therefore, more than half of world’s new cases and deaths from EC occur in China. Recently, despite great efforts in therapeutic measures, the treatment outcomes for patients with EC remain unsatisfactory (3, 4). The main reasons for poor prognosis are late diagnosis, regional and distant metastasis, treatment resistance and frequent recurrence (5–7). Thus, it is urgent to identify more effective clinical indicators to predict prognosis and improve survival prior to treatment in patients with EC.

Accumulating studies have indicated that nutritional and/or inflammatory status was closely correlated to prognosis in a variety of cancers (8, 9). Recently, preoperative hematological inflammation and nutrition based indicators, such as albumin (ALB), C-reactive protein (CRP), platelet (PLT) to lymphocyte (LYM) ratio (PLR), neutrophil (NEU) to LYM ratio (NLR), and LYM to monocyte (MON) ratio (LMR), have demonstrated their prognostic roles in various cancers (10–13). Moreover, researchers were not satisfied with the single indicator and sought to more and more integrative indicators. A variety of studies published in recent years also revealed that several integrative indicators, such as systemic immune-inflammation index (SII), systemic inflammation response index (SIRI), and prognostic nutritional index (PNI) were prognostic factors for cancer prognosis (14–16). However, the results for these above indexes are still controversial in cancers. Moreover, the hematological inflammation and nutrition based indicators mentioned above may be affected by various other factors.

It is indicated that the combination of these indicators may provide more accurate prognostic values than individual indicators. Moreover, the influence of confounding factors may be reduced by the combination of these indicators. In addition, accurate prognosis prediction may help clinician choose appropriate therapeutic treatments based on risk stratification. Therefore, we initially developed and verified an integrative inflammatory and nutritional score (IINS) by using least absolute shrinkage and selection operator (LASSO) to predict clinical outcomes in ESCC patients.

## Materials and methods

### Patient selection

A series of 672 pathologically confirmed EC patients undergoing radical resection in our institute were retrospectively collected from 2012 to 2014. The inclusion criteria were (1) ESCC histologically confirmed; (2) patients with radical resection (R0 resection); (3) patients with full medical records and follow-up; (4) patients with no preoperative therapy; (5) patients with no acute or chronic infection, autoimmune disease or hematologic disease; and (6) patients with no other previous or synchronous malignancy. As a retrospective study with anonymous data, informed consent was waived. Study approval was obtained from the ethics committee of Zhejiang Cancer Hospital (Number: 2021-5). The study was performed in line with the Declaration of Helsinki.

### Treatment measures and follow-up

The main surgical procedure in the present study included McKeown or Ivor Lewis with two or three-field lymphadenectomy (17). The stage in the current study was in accordance with the 8th AJCC/UICC tumor node metastasis (TNM) staging system (18). Neoadjuvant treatments were recommend by the NCCN guidelines for locally advanced ESCC, however, a large number of ESCC patients in China tended to choose surgery as initial treatment (19, 20). Adjuvant therapy was mainly carried out based on the post-operative pathological results (21). Generally speaking, if the post-operative pathology result indicated stage T3 or higher and/or lymph node metastasis, cisplatin-based chemotherapy and/or radiotherapy would be administered (22, 23). Patients were then followed up including physical examinations, tumor markers tests, and contrast CT examinations with regular checks (first 2 years: quarterly; next 2–5 years: semiannually; after 5 years: annually). The last follow-up date for the patients was Dec. 2019.

### Integrative inflammatory and nutritional score definition and other conventional scores

The clinical data including preoperative hematological indexes were retrospectively collated. The hematological indicators, such as CRP, ALB, NEU, LYM, PLT, NLR, PLR, LMR, hemoglobin (HB), PLT to HB ratio (PHR), NEU to HB ratio (NHR), CRP to HB ratio (CHR), CRP to prealbumin (PALB) ratio (CPR), total protein (TP), globulin (GLO), and so on, were tested within 1 week before the surgery. The other variables (SII, SIRI, and PNI) were calculated by the following formula based on published studies: PNI = ALB (g/L) + 5 × LYM (10^9^/L), SII = PLT × NEU/LYM, and SIRI = MON × NEU/LYM (14–16).

### Statistical analysis

All data were analyzed in the current study by using R software (version 3.6.1), SPSS 20.0 and Medcalc 17.6. The LASSO Cox PH regression analysis was used to select useful indicators. The “maxstat” package of R software was used to classify various inflammatory and nutritional indicators based on the cutoff point determined by the maximally selected rank statistics. The “glmnet” package of R software was performed to identify the most valuable prognostic factors among all candidate hematological inflammatory and nutritional indicators by LASSO Cox PH regression analysis. In order to shrink some regression coefficients to exactly zero, an L1 penalty was set in the LASSO model. Then 10-fold cross-validation with minimum criteria was carried out to find the optimal log (λ). These above hematological inflammatory and nutritional indicators with non-zero coefficients were incorporated to construct the novel score of IINS, which was calculated as follows: IINS = sum (score of every indicator × relevant regression coefficients from LASSO model). The optimum cut-off value for IINS in our study was calculated by the Cutoff Finder^1^ with the method of survival: significance (log-rank test), which was a straightforward and comprehensive application enabling rapid cutoff optimization (24). According to the Medcalc 17.6 statistical software with DeLong’s methods, the areas under the curve (AUCs) and the 95% confidence intervals (CIs) between IINS and SIRI, SII, and PNI were compared by the receiver operating characteristic (ROC) curves (25, 26). Independent prognostic factors for cancer-specific survival (CSS) were identified by Cox PH regression analyses with hazard ratio (HRs) and 95% CIs. The curves of CSS were calculated by using Kaplan–Meier method. The CSS was defined as the time between surgery and death resulting from the primary cancer according to previous published study (27). Finally, a novel nomogram model was also established to verify the prognostic value of independent indicators. Subsequently, the predictive accuracy and discriminative ability were assessed by the calibration curves, ROC curves and decision curve analyses (DCA). A two-side *P*-value < 0.05 was considered to be statistically significant.

## Results

### Patient characteristics

**FIGURE 1:**
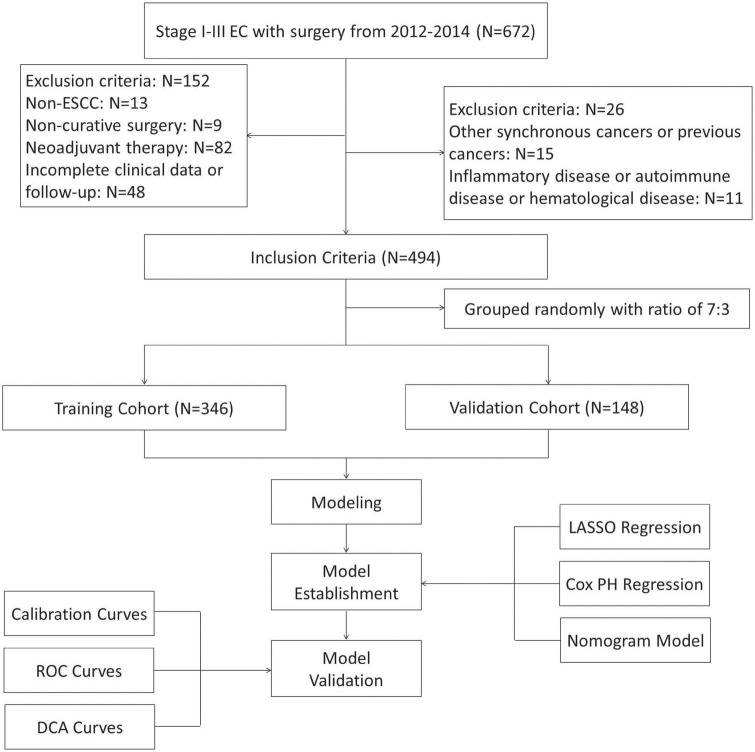
The flow diagram of selection esophageal squamous cell carcinoma (ESCC) patients with radical resection. Based on the inclusion and exclusion criteria, a total of 494 patients were randomly included.

A total of 494 patients were enrolled in the current study (training set: *n* = 346 and validation set: *n* = 148) (Figure 1). There was no statistically significant difference in mean age between the two groups (training cohort: 59.1 ± 7.5 years and validation cohort: 60.0 ± 6.6 years; *P* = 0.178). However, there was a sex difference between the two groups (*P* = 0.033). Regarding to the inflammatory and nutritional indexes, the values of TP were significant higher in the training set than those in validation set (7.21 ± 0.53 g/dL vs. 7.09 ± 0.52 g/dL, *P* = 0.019), while the values of PALB were significant lower in training group than those in the validation group (256.9 ± 64.2 mg/L vs. 270.5 ± 64.1 mg/L, *P* = 0.031). The baseline clinicopathologic features between these two groups were displayed in Table 1.

**TABLE 1:** Comparison of the baseline characteristics in the training and validation cohorts.

	Training set (*n* = 346)	Validation set (*n* = 148)	*P*-value
Age (mean ± SD, years)	59.1 ± 7.5	60.0 ± 6.6	0.178
Sex (male/female)	236/110	115/33	0.033
Tumor length (mean ± SD, cm)	4.21 ± 1.79	4.28 ± 1.65	0.660
Tumor location (upper/middle/lower)	20/158/168	12/62/74	0.537
Vessel invasion (no/yes)	286/60	122/26	0.951
Perineural invasion (no/yes)	272/74	124/24	0.187
Differentiation (well/moderate/poor)	53/228/65	21/95/32	0.756
TNM stage (I/II/III)	99/120/127	48/55/45	0.393
Adjuvant treatment (no/yes)	247/99	114/34	0.195
**Inflammatory and nutritional indexes**			
NEU (mean ± SD, 10^9/L)	4.44 ± 1.51	4.49 ± 1.64	0.712
LYM (mean ± SD, 10^9/L)	1.59 ± 0.50	1.54 ± 0.41	0.235
MON (mean ± SD, 10^9/L)	0.54 ± 0.20	0.51 ± 0.13	0.177
PLT (mean ± SD, 10^9/L)	228.0 ± 70.1	224.4 ± 72.2	0.600
HB (mean ± SD, g/L)	123.2 ± 12.5	124.3 ± 11.9	0.359
CRP (mean ± SD, mg/L)	7.09 ± 8.35	8.51 ± 8.01	0.081
ALB (mean ± SD, g/dL)	4.08 ± 0.51	4.05 ± 0.56	0.532
GLO (mean ± SD, g/dL)	3.13 ± 0.67	3.06 ± 0.67	0.315
TP (mean ± SD, g/dL)	7.21 ± 0.53	7.09 ± 0.52	0.019
PALB (mean ± SD, mg/L)	256.9 ± 64.2	270.5 ± 64.1	0.031
LDH (mean ± SD, U/L)	180.0 ± 55.8	181.3 ± 78.6	0.840
PHR (mean ± SD)	1.86 ± 0.58	1.81 ± 0.57	0.393
NHR (mean ± SD)	0.036 ± 0.013	0.037 ± 0.014	0.891
CHR (mean ± SD)	0.058 ± 0.068	0.069 ± 0.066	0.103
PLR (mean ± SD)	154.2 ± 60.9	156.0 ± 64.8	0.777
NLR (mean ± SD)	2.93 ± 1.06	3.06 ± 1.22	0.235
CLR (mean ± SD)	4.86 ± 6.28	6.03 ± 5.95	0.055
LMR (mean ± SD)	3.10 ± 0.71	3.06 ± 0.63	0.587
CAR (mean ± SD)	1.83 ± 2.34	2.24 ± 2.34	0.077
CPR (mean ± SD)	0.029 ± 0.034	0.032 ± 0.032	0.260
AGR (mean ± SD)	1.40 ± 0.48	1.42 ± 0.51	0.587
LAR (mean ± SD)	44.71 ± 14.62	45.62 ± 21.62	0.590
LPR (mean ± SD)	0.75 ± 0.32	0.73 ± 0.39	0.412
PNI (mean ± SD)	48.7 ± 5.4	48.1 ± 5.9	0.276
SII (mean ± SD)	679.1 ± 340.4	707.4 ± 392.0	0.420
SIRI (mean ± SD)	1.50 ± 0.61	1.54 ± 0.69	0.595
IINS (mean ± SD)	2.33 ± 0.54	2.34 ± 0.55	0.978

SD, standard deviation; NEU, neutrophil; LYM, lymphocyte; MON, monocyte; PLT, platelet; CRP, C-reactive protein; HB, hemoglobin; LDH, lactate dehydrogenase; TP, total protein; GLO, globulin; ALB, albumin; PALB, prealbumin; CAR, CRP to ALB ratio; CPR, CRP to PALB ratio; CHR, CRP to HB ratio; CLR, CRP to LYMPH ratio; NLR, NEUT to LYMPH ratio; NHR, NEUT to HB ratio; PLR, PLT to LYMPH ratio; PHR, PLT to HB ratio; LMR, LYMPH to MONO ratio; LAR, LDH to ALB ratio; LPR, LDH to PALB ratio; TNM, tumor node metastasis; PNI, prognostic nutritional index; SII, systemic immune-inflammation index; SIRI, systemic inflammation response index; IINS, integrative inflammatory and nutritional score.

### Integrative inflammatory and nutritional score construction based on inflammatory and nutritional indicators

**FIGURE 2:**
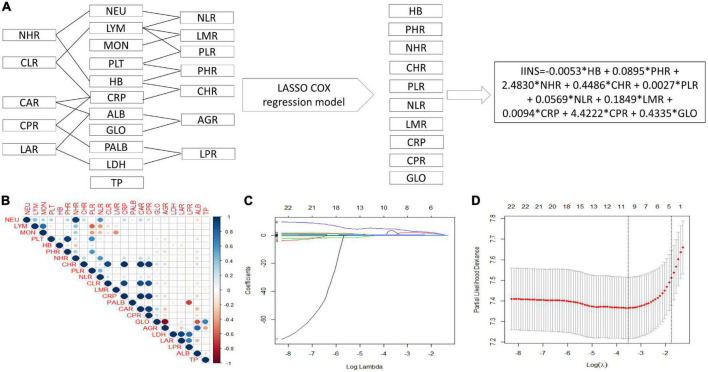
Process diagram for integrative inflammatory and nutritional score (IINS) construction and risk stratification. IINS constructed with 10 indicators out of 23 variables by least absolute shrinkage and selection operator (LASSO) Cox proportional hazards (PH) regression model **(A)**. A correlation matrix is represented regarding 23 indicators **(B)**. LASSO coefficient profiles of the 23 indicators **(C)**. 10-fold cross–validation for tuning parameter selection in the LASSO model **(D)**.

The process diagram of IINS was shown in Figure 2A. The correlation of heat map for 23 inflammatory and nutritional indicators was shown in Figure 2B. According to the LASSO Cox PH regression model, 10 indicators including HB, PHR, NHR, CHR, PLR, NLR, LMR, CRP, CPR, and GLO were selected out of 23 inflammatory and nutritional indexes (Figures 2C,D). Finally, the IINS = –0.0053 × HB + 0.0895 × PHR + 2.4830 × NHR + 0.4486 × CHR + 0.0027 × PLR + 0.0569 × NLR + 0.1849 × LMR + 0.0094 × CRP + 4.4222 × CPR + 0.4335 × GLO.

### Areas under the curve comparisons between integrative inflammatory and nutritional score and other conventional indexes (systemic inflammation response index, systemic immune-inflammation index, and prognostic nutritional index)

**FIGURE 3:**
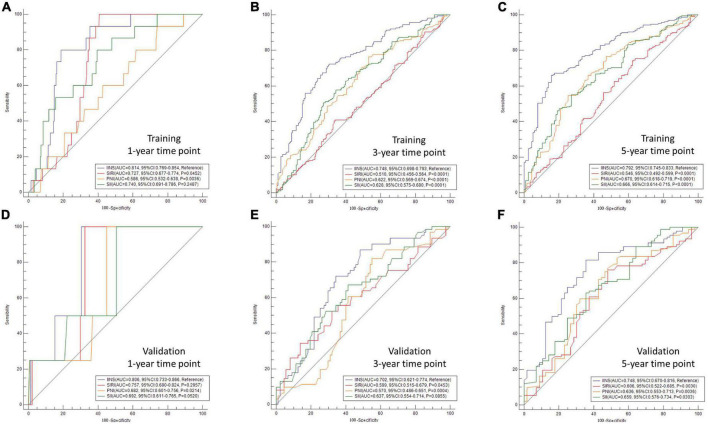
Areas under the curve (AUC) comparisons in different time points between integrative inflammatory and nutritional score (IINS) and other variables. AUC comparisons in different time points were compared between IINS and other conventional indexes [systemic inflammation response index (SIRI), systemic immune-inflammation index (SII), and prognostic nutritional index (PNI)]. The AUCs of IINS regarding prognostic ability in 1-year, 3-years, and 5-years prediction were 0.814 **(A)**, 0.748 **(B)**, and 0.792 **(C)** in the training cohort and 0.802 **(D)**, 0.702 **(E)**, and 0.748 **(F)** in the validation cohort, respectively.

To better understand the predictive ability between IINS and other conventional established prognostic scores, AUC comparisons in different time points (1-year, 3-years, and 5-years) were compared between IINS and other conventional indexes (SIRI, SII, and PNI) (Figure 3). The AUCs of IINS regarding prognostic ability in 1-year, 3-years, and 5-years prediction were 0.814 (95% CI: 0.769–0.854), 0.748 (95% CI: 0.698–0.793), and 0.792 (95% CI: 0.745–0.833) in the training cohort and 0.802 (95% CI: 0.733–0.866), 0.702 (95% CI: 0.621–0.774), and 0.748 (95% CI: 0.670–0.816) in the validation cohort, respectively. Therefore, IINS had the largest AUCs in the training cohort and validation cohort compared with other prognostic indicators (SIRI, PNI, and SII), indicating a higher predictive ability.

### Patient characteristics grouped by integrative inflammatory and nutritional score

**FIGURE 4:**
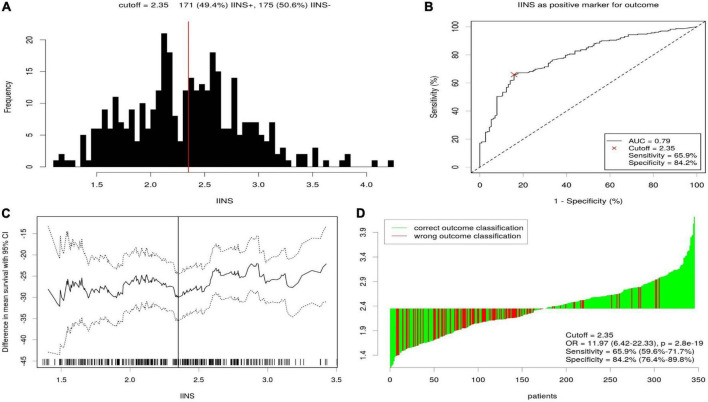
The optimal cutoff value achieved for integrative inflammatory and nutritional score (IINS). Distribution for IINS based cutoff optimization **(A)**. A score of 2.35 was chosen as the optimal cutoff point by receiver operating characteristic (ROC) **(B)**. Cutoff optimization by correlation with cancer-specific survival (CSS) prediction **(C)**. Waterfall plot for IINS **(D)**.

**FIGURE 5:**
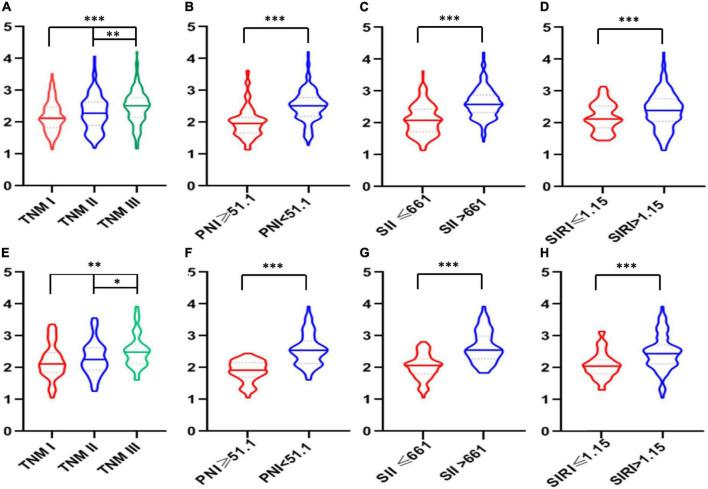
The violin plots of integrative inflammatory and nutritional score (IINS) values. The violin plots of IINS grouped by tumor node metastasis (TNM), prognostic nutritional index (PNI), systemic immune-inflammation index (SII), and systemic inflammation response index (SIRI) in both training cohort **(A–D)** and validation cohort **(E–H)**.

According to the cutoff finder and ROC curve, the cut-off level of IINS in the training set was 2.35 (Figure 4). Patients were divided into two groups for further analysis with the cut-off value of 2.35. IINS was significantly associated with vessel and perineural invasion, TNM stage, SIRI, PNI, and SII in both training cohort and validation cohort. Tumor length was significantly associated with IINS in the training set (*P* < 0.001), but not in the validation set (*P* = 0.095) (Table 2). In addition, the values regarding IINS grouped by TNM stage, SIRI, PNI, and SII in these two groups were also displayed in Figure 5.

**TABLE 2:** Comparison of baseline characteristics based on integrative inflammatory and nutritional score (IINS) in training and validation sets.

	Training			Validation		
	IINS ≤ 2.35	IINS > 2.35	*P*-value	IINS ≤ 2.35	IINS > 2.35	*P*-value
Age (years, ≤60/>60)	106/69	101/70	0.775	40/42	42/24	0.071
Sex (male/female)	118/57	118/53	0.753	60/22	55/11	0.140
Tumor length (cm, ≤3.0/>3.0)	71/104	32/139	<0.001	29/53	15/51	0.095
Tumor location (U/M/L)	13/77/85	7/81/83	0.391	6/38/38	6/24/36	0.472
Vessel invasion (no/yes)	152/23	134/37	0.037	73/9	49/17	0.019
Perineural invasion (no/yes)	148/27	124/47	0.006	75/7	49/17	0.005
Differentiation (W/M/P)	28/112/35	25/116/30	0.749	14/55/13	7/40/19	0.126
TNM stage (I/II/III)	67/63/45	32/57/82	<0.001	32/33/17	16/22/28	0.014
Adjuvant treatment (no/yes)	126/49	121/50	0.799	62/20	52/14	0.648
SIRI (≤1.15/>1.15)	63/112	41/130	0.015	41/41	10/56	< 0.001
PNI (≥51.1/<51.1)	100/75	17/154	<0.001	48/34	2/64	< 0.001
SII (≤661/>661)	131/44	56/115	<0.001	59/23	14/52	< 0.001

TNM, tumor node metastasis; PNI, prognostic nutritional index; SII, systemic immune-inflammation index; SIRI, systemic inflammation response index; IINS, integrative inflammatory and nutritional score; U/M/L, upper/middle/lower; W/M/P, well/moderate/poor.

### Kaplan–Meier analyses of cancer-specific survival and Cox proportional hazards regression analyses

**FIGURE 6:**
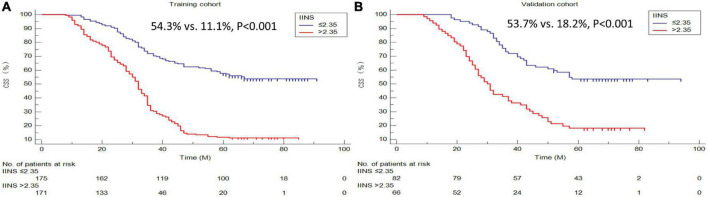
Cancer-specific survival (CSS) analyses grouped by integrative inflammatory and nutritional score (IINS). Kaplan–Meier curves regarding 5-years CSS in the training cohort **(A)** and validation cohort **(B)**.

**FIGURE 7:**
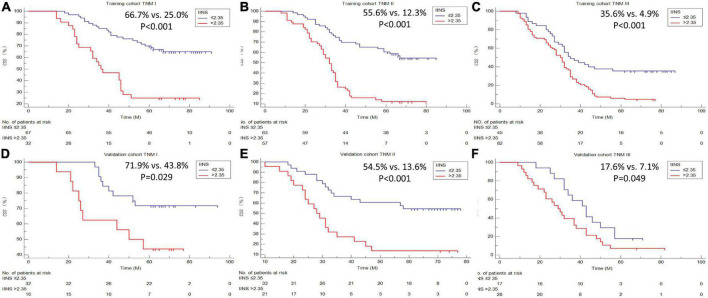
Cancer-specific survival (CSS) analyses grouped by tumor node metastasis (TNM) stages. Subgroup analyses based on TNM stage in the training cohort **(A–C)** and validation cohort **(D–F)**.

A better 5-years CSS was found in patients with IINS ≤ 2.35 compared with those with IINS > 2.35 in both training cohort (54.3% vs. 11.1%, *P* < 0.001) and validation cohort (53.7% vs. 18.2%, *P* < 0.001) (Figure 6). Subgroup analyses suggested that IINS (training or validation cohort) had reliable abilities to predict prognosis in resected ESCC patients in any TNM stages (Figure 7). The IINS was confirmed as a useful independent factor [training set: (HR 3.000, 95% CI 2.254–3.992, *P* < 0.001); validation set: HR 2.609, 95% CI 1.693–4.020, *P* < 0.001] (Tables 3, 4).

**TABLE 3:** Univariate and multivariate Cox analyses of cancer-specific survival (CSS) in training set.

	Univariate analyses		Multivariate analyses	
	HR (95% CI)	*P*-value	HR (95% CI)	*P*-value
Age (years, >60/≤60)	0.977 (0.751–1.270)	0.861		
Sex (male/female)	0.907 (0.690–1.194)	0.487		
Tumor length (cm, >3.0/≤3.0)	1.306 (0.980–1.740)	0.068		
**Tumor location**				
Middle/upper	1.261 (0.694–2.290)	0.447		
Lower/upper	1.269 (0.700–2.300)	0.433		
Vessel invasion (yes/no)	1.674 (1.218–2.302)	0.002		
Perineural invasion (yes/no)	1.643 (1.224–2.206)	0.001		
**Differentiation**				
Moderate/well	1.152 (0.791–1.678)	0.460		
Poor/well	1.322 (0.842–2.076)	0.226		
**TNM stage**				
II/I	1.694 (1.179–2.434)	0.004	1.534 (1.066–2.207)	0.021
III/I	2.996 (2.120–4.235)	< 0.001	2.206 (1.548–3.146)	< 0.001
Adjuvant treatment (yes/no)	1.067 (0.804–1.415)	0.654		
PNI (<51.1/≥51.1)	2.145 (1.588–2.899)	< 0.001		
SII (>661/≤661)	1.997 (1.539–2.591)	< 0.001		
SIRI (>1.15/≤1.15)	1.388 (1.036–1.859)	0.028		
IINS (>2.35/≤2.35)	3.470 (2.629–4.581)	< 0.001	3.000 (2.254–3.992)	< 0.001

CSS, cancer-specific survival; PNI, prognostic nutritional index; SII, systemic immune-inflammation index; IINS, integrative inflammatory and nutritional score; SIRI, systemic inflammation response index; HR, hazard ratio; CI, confidence interval; TNM, tumor node metastasis.

**TABLE 4:** Univariate and multivariate Cox analyses of cancer-specific survival (CSS) in validation set.

	Univariate analyses		Multivariate analyses	
	HR (95% CI)	*P*-value	HR (95% CI)	*P*-value
Age (years, >60/≤60)	0.994 (0.659–1.499)	0.976		
Sex (male/female)	1.216 (0.726–2.035)	0.457		
Tumor length (cm, >3.0/≤3.0)	1.143 (0.722–1.810)	0.569		
**Tumor location**				
Middle/upper	0.672 (0.337–1.343)	0.261		
Lower/upper	0.471 (0.235–0.947)	0.035		
Vessel invasion (yes/no)	1.894 (1.171–3.062)	0.009		
Perineural invasion (yes/no)	1.806 (1.088–2.997)	0.022		
**Differentiation**				
Moderate/well	0.951 (0.521–1.736)	0.871		
Poor/well	1.318 (0.660–2.634)	0.434		
**TNM stage**				
II/I	2.218 (1.251–3.932)	0.006	2.271 (1.279–4.031)	0.005
III/I	3.921 (2.236–6.975)	< 0.001	3.181 (1.799–5.625)	< 0.001
Adjuvant treatment (yes/no)	1.041 (0.645–1.680)	0.870		
PNI (<51.1/≥51.1)	2.510 (1.540–4.092)	< 0.001		
SII (>661/≤661)	1.973 (1.298–2.998)	0.001		
SIRI (>1.15/≤1.15)	1.908 (1.189–3.062)	0.007		
IINS (>2.35/≤2.35)	2.975 (1.957–4.523)	< 0.001	2.609 (1.693–4.020)	< 0.001

CSS, cancer-specific survival; PNI, prognostic nutritional index; SII, systemic immune-inflammation index; IINS, integrative inflammatory and nutritional score; SIRI, systemic inflammation response index; HR, hazard ratio; CI, confidence interval; TNM, tumor node metastasis.

### Nomogram development and validation

**FIGURE 8:**
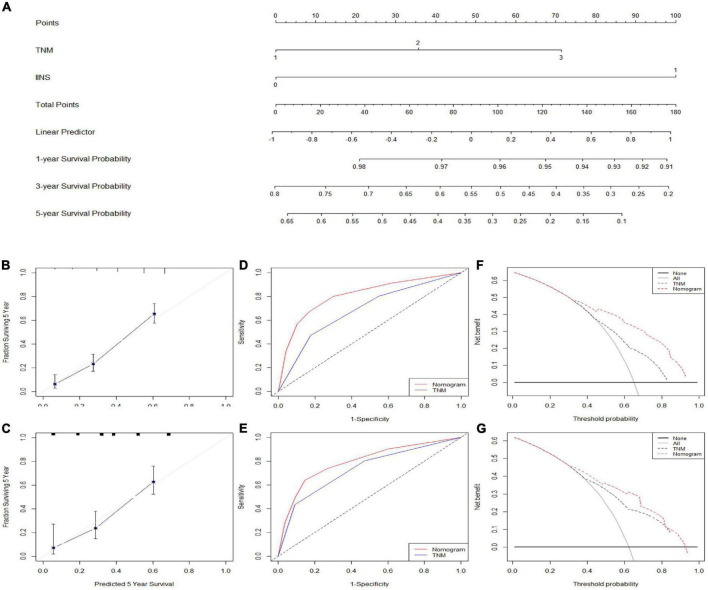
Nomogram established and validated. Nomogram established based on integrative inflammatory and nutritional score (IINS) and tumor node metastasis (TNM) **(A)**. Calibration curves for 5-years cancer-specific survival (CSS) prediction in the training cohort **(B)** and validation cohort **(C)**. Receiver operating characteristic (ROC) analyses of CSS prediction in the training cohort **(D)** and validation cohort **(E)**. Decision curve analyses (DCA) revealed nomogram model in the training cohort **(F)** and validation cohort **(G)**. TNM 1/2/3, stage I/II/III; IINS 0/1, ≤2.35/>2.35.

A nomogram model (training set: C-index = 0.71 and validation set: C-index = 0.69) based on IINS and TNM stage, two independent significant variables in multivariate analyses, was established to predict individual CSS in ESCC (Figure 8A). An acceptable agreement was represented in calibration curves for 5-years CSS in both two cohorts (Figures 8B,C). Compared with traditional TNM stages, the predictive accuracy and discriminative ability for ROC (Figures 8D,E) and DCA (Figures 8F,G) was much better in both two cohorts. All these results in our study indicated that the current nomogram model based on IINS showed excellent risk stratification and prognostic accuracy.

## Discussion

In this study, we initial developed and verified a novel prognostic score of IINS based on various preoperative hematological indexes in patients with ESCC. Our results confirmed the prognostic effect of IINS as a novel indicator in patients with ESCC. IINS had the largest AUCs both in the training cohort and validation cohort compared with other indicators, indicating a higher predictive ability for prognostic prediction in ESCC. Subsequently, a novel predictive nomogram based on two variables (IINS and TNM) was firstly established and validated, showing excellent risk stratification and prognostic accuracy.

To date, determining the prognosis of ESCC patients before treatment has been a challenge. Therefore, it is very important to identify the prognostic value of the clinical characteristics as well as various hematological indicators in cancers. Based on these prognostic analyses, clinicians may make their further risk stratification and formulate individualized therapeutic strategy. It is indicated in several studies that inflammation and nutrition is associated with cancer prognosis (10–16). Inflammatory and nutritional status is closely associated with carcinogenesis, cancer growth and progression (8, 9). Moreover, prognostic values of several preoperative inflammatory and nutritional indicators have been explored in ESCC patients in the past few years (13, 14, 16). A meta-analysis conducted by Ishibashi et al. (13) aimed to explore the correlations between PLT associated markers and overall survival (OS) in ESCC. Based on 14 retrospective studies, they demonstrated that low PLR level was significantly associated with well OS. Fujiwara et al. (14) analyzed several indexes based on inflammation and nutrition in 111 ESCC patients and the results revealed low PNI was associated with shorter recurrence-free survival (RFS). Geng et al. (16) revealed that SIRI was significantly related to OS and served as an independent prognostic score in 916 ESCC patients with radical resection.

In fact, a single inflammatory and nutritional indicator has some limitations and can’t fully reflect the overall status of inflammation and nutrition (28). Moreover, most of published studies incorporated these inflammatory and nutritional indicators in multivariate analyses to explore independent prognostic factors (14–16), but ignore the strong collinearity and correlation between these indicators, causing variables interference and some statistical problems. In addition, an integrated prognostic indicator consisting of multiple dimensions may help to reflect the real and complicated inflammatory and nutritional status (29). In the face of so many features, it is very important to eliminate over-fitting in feature selection (30, 31). Published studies revealed that over-fitting might be solved by applying bootstrapping technique and LASSO Cox PH regression analysis (32). Recently, several studies established some prognostic models with a variety of hematological indicators by LASSO regression. Wang et al. (33) established an inflammatory and nutritional prognostic score (INPS) based on 15 hematological indexes in 513 stage III gastric cancer. They confirmed that INPS was a prognostic factor. They also conducted a nomogram based on INPS for survival prediction in resected stage III gastric cancer with adjuvant chemotherapy. Hua et al. (34) also validated the prognostic value of INPS by LASSO regression analysis in 1,259 patients with early stage breast cancer. In another study including 334 patients with limited-stage small cell lung cancer, the authors also indicated that several hematological nutritional and inflammatory indexes were associated with OS (35). This study conducted an integrated score by LASSO Cox PH regression model to effectively select valuable variables, reducing the influence of multi-collinearity to some extent.

There were several possible mechanisms to illustrate the relationships between cancer and inflammation-nutrition. Most published studies revealed that various inflammatory markers, such as NEU, MON, LYM, PLT, and CRP, play a very important role in promoting metastasis of cancer cells, increasing vascular proliferation and permeability, regulating cancer progression and metastasis, promoting immune surveillance, and antitumor immune response (36–39). The mechanism regarding nutritional markers, such as HB, ALB, GLO, and PALB, in cancer include regulating hypoxia of cancer cells, stimulating cancer growth and progression, activating a variety of cytokines, such as tumor necrosis factor-α and interleukin-1, and increasing the resistance to chemotherapy and/or radiotherapy (40–42).

Nomogram is considered to be a reliable tool for integrating and quantifying significant risk factors for cancer prognosis (43). Nomogram, as a prognostic statistical model, can not only visually display the relevant indicators affecting the outcome in Cox PH regression analyses but also predict the survival probability through a simple graphical representation, which makes the prediction more simple and convenient (44). Wang et al. (33) and Hua et al. (34) both established a nomogram based on INPS to predict prognosis. They also revealed that the INPS-based prognostic nomogram showed a good prognostic stratification. Mao et al. (45) developed and verified a nomogram in hepatocellular carcinoma patients after surgical resection. They indicated that nomogram model may guide clinical prediction, personalized treatment approach and prognosis. In the current study, a novel predictive nomogram based on IINS and TNM was firstly performed and validated in ESCC, which showed excellent risk stratification and prognostic accuracy.

Our study had some limitations that should be noticed. Firstly, this was a single-center retrospective study. So there may be potential data collection bias. As a retrospective design, all data in the current study were observational statistics and were subject to selection bias inherent in non-randomized retrospective designs (46, 47). It is possible that some cases (particularly for those without full medical records and/or follow-up time) could have been missed. Moreover, although we have excluded immune diseases, hematological diseases, and inflammatory diseases, other diseases such as essential hypertension and diabetes mellitus might also affect these blood indicators (48, 49). Secondly, other factors, such as genomics and tumor biomarkers, and coagulation indicators, influencing the ESCC prognosis were not included in the construction of IINS. Thirdly, hematological inflammatory and nutritional variables may be affected by other factors and the results may be changed. Fourthly, the results may be biased due to the lack of additional independent external validation cohort. Fifthly, the exact mechanisms regarding inflammation and nutrition in cancer prognosis require further explore. Sixthly, it would be more convincing if the follow-up time could be updated to the present. However, patient follow-up is a long-term and onerous task, which requires a lot of manpower and material resources, so we can’t get the latest follow-up data in a short time. Last but not least, the prognostic value of IINS needs to be confirmed in various other cancers. Although the above limitations existed, our developed IINS-based nomogram may be used as a potential risk stratification to predict individual CSS in ESCC.

## Conclusion

In summary, the preoperative IINS is an independent predictor of CSS in ESCC patients with radical resection. The nomogram based on IINS may be used as a potential risk stratification to predict individual CSS and guide treatment in ESCC with radical resection.

## Data availability statement

The original contributions presented in the study are included in the article/supplementary material, further inquiries can be directed to the corresponding authors.

## Ethics statement

The studies involving human participants were reviewed and approved by the study was performed in line with the Declaration of Helsinki. As a retrospective study with anonymous data, informed consent was waived. Study approval was obtained from the Ethics Committee of Zhejiang Cancer Hospital (Number: 2021-5). Written informed consent for participation was not required for this study in accordance with the national legislation and the institutional requirements.

## Author contributions

JF, QC, and XC contributed to the study design and prepared for the manuscript. LW and XY contributed to the data collect. JF, LW, and XY contributed to the statistical analysis. All authors approved the final manuscript as submitted and agreed to be accountable for all aspects of the work.

## Footnotes

1
http://molpath.charite.de/cutoff/
